# Effects of age, sex, and sensory information on balance performance in young and older adults

**DOI:** 10.3389/fragi.2026.1761492

**Published:** 2026-02-09

**Authors:** Ahmad Ali Akbari Kamrani, Amir Shams, Parvaneh Shamsipour Dehkordi, Robab Sahaf, Mahdi Bayati, Hamed Abbasi, Urs Granacher, Lara Carneiro

**Affiliations:** 1 Department of Aging, Iranian Research Center on Aging, University of Social Welfare and Rehabilitation Sciences, Tehran, Iran; 2 Department of Motor Behavior, Sport Sciences Research Institute, Tehran, Iran; 3 Department of Motor Behavior, Faculty of Sport Sciences, Alzahra University, Tehran, Iran; 4 Department of Aging, Iranian Research Center on Aging, University of Social Welfare and Rehabilitation Sciences, Tehran, Iran; 5 Department of Exercise Physiology, Sport Sciences Research Institute, Tehran, Iran; 6 Department of Sports Medicine, Sport Sciences Research Institute, Tehran, Iran; 7 Department of Sport and Sport Science, Exercise and Human Movement Science, University of Freiburg, Freiburg, Germany; 8 Physical Education Department, College of Education, United Arab Emirates University, Abu Dhabi, United Arab Emirates

**Keywords:** aging, center of pressure, postural control, sensory integration, sex differences

## Abstract

**Background:**

This study aimed to comprehensively investigate the independent and interactive effects of age, sex, and sensory information on balance control in young and older adults.

**Methods:**

A total of 250 participants, stratified into five age groups (25–40, 60–65, 66–70, 71–75, and 76–80 years) with equal sex distribution, underwent the Sensory Organization Test (SOT) using computerized dynamic posturography. Balance was assessed using center of pressure (COP) velocity and displacement across six sensory conditions that selectively challenged and altered the availability and reliability of visual, proprioceptive, and vestibular information, thereby eliciting adaptive multisensory reweighting rather than isolating individual sensory systems. A 5 (age group) × 2 (sex) × 6 (sensory condition) repeated-measures ANOVA was used for analysis.

**Results:**

The analysis revealed significant main effects of age group and sensory condition on both COP velocity and displacement (age group: p < 0.001 for all; sensory condition: p < 0.001 for all), with balance performance systematically declining with each successive age group and as sensory conditions became more challenging. No significant main sex effects were found. Critically, significant interactions revealed that the detrimental effects of age and sensory conditions were not uniform across all groups. Notably, the effect of challenging sensory conditions was more pronounced in older adults (age × condition, p < 0.001, *d* = 0.50). Furthermore, a significant age × sex interaction (p = 0.001, *d* = 0.59) indicated that sex differences emerged primarily in the oldest cohort (76–80 years), where females exhibited greater instability than males.

**Conclusion:**

Balance control is profoundly influenced by age and the availability of accurate sensory information, with older adults, especially the oldest old, demonstrating significantly greater impairment under sensory-challenging conditions. While sex alone was not a dominant factor, its interaction with age suggests that the oldest females may represent a particularly vulnerable subgroup.

## 1 Introduction

Aging is accompanied by progressive deterioration in sensory, motor, neural, and cognitive systems, all of which play critical and interdependent roles in postural control. As a result, balance impairments become increasingly prevalent with advancing age, substantially limiting the ability of older adults to perform daily activities and maintain functional independence. Given the rapidly growing global population of older adults and the profound personal, clinical, and socioeconomic consequences of falls, identifying the mechanisms underlying age-related postural instability has emerged as a major public health priority (Kim et al., 2010; Roman-Liu, 2018; Røgind et al., 2003; Era et al., 2006).

Postural control depends on the dynamic integration of visual, proprioceptive, and vestibular inputs. With advancing age, not only do individual sensory systems decline, but the central nervous system’s ability to flexibly reweight sensory information under conditions of sensory conflict or deprivation is also impaired (Jones and Noppeney, 2021; Behtani et al., 2023). Consequently, experimental paradigms that systematically manipulate sensory inputs are particularly valuable for detecting age-related deficits in balance control that may not be evident under stable or unchallenged conditions (Behtani et al., 2023; Palazzo et al., 2021).

Beyond the effects of aging alone, sex-related differences represent another important yet incompletely understood dimension of postural control. In young adults, most studies report minimal or no significant sex differences in postural stability during quiet standing (Olchowik et al., 2015; Sell et al., 2018; Greve et al., 2013; Błaszczyk et al., 2014). However, findings in older populations are inconsistent, with several investigations reporting lower postural sway in older women compared with men (Kim et al., 2010; Puszczalowska-Lizis et al., 2018; Bryant et al., 2005; Masui et al., 2005; Era et al., 1996; Wiśniowska-Szurlej et al., 2019), whereas others suggest superior balance performance in older men or no significant sex difference (Hageman et al., 1995; Nakagawa et al., 2017; Šarabon et al., 2022). These discrepancies have been attributed to multiple factors, including anthropometric characteristics such as height and body mass (Bryant et al., 2005; Era et al., 1996), age- and sex-related differences in muscle strength and physical capacity (Cooper et al., 2011; Frontera et al., 1985; Cahalan et al., 1989), and potential differences in sensory integration strategies between men and women (Masui et al., 2005; Faraldo-García et al., 2012). Importantly, growing evidence indicates that such sex-related differences in balance control may become more pronounced under sensory-challenged conditions. Older adults tend to rely more heavily on visual input for postural stability, and this reliance may differ between men and women, particularly when visual or proprioceptive information is degraded (Masui et al., 2005; Faraldo-García et al., 2012). In contrast, vestibular contributions to balance appear to be less influenced by sex, although their interaction with aging remains incompletely understood (Faraldo-García et al., 2012).

Despite a growing body of research, a critical gap remains. Many previous studies have examined the effects of age or sex in isolation, relied on subjective or low-sensitivity clinical balance tests, or assessed postural control under a limited number of sensory conditions. Few investigations have systematically evaluated the independent and interactive effects of age and sex across multiple sensory manipulation conditions within a single standardized experimental framework. This limitation contributes to inconsistent findings and hinders a comprehensive understanding of multisensory balance control across the adult lifespan.

Instrumented assessments provide a more sensitive and objective evaluation of postural control. Among these, Computerized Dynamic Posturography (CDP), particularly the Sensory Organization Test (SOT), is widely regarded as a gold-standard method for assessing functional balance and multisensory integration by systematically altering visual and proprioceptive inputs while quantifying center-of-pressure responses (Wafa et al., 2023).

Therefore, the primary aim of this study was to investigate balance performance across young and older adults by examining the independent and interactive effects of age, sex, and sensory condition using CDP-derived measures of center-of-pressure velocity and displacement. By including multiple older-age cohorts and a range of sensory manipulation conditions, this study seeks to clarify how aging and sex jointly influence multisensory balance control. Based on existing evidence, we hypothesized that balance performance would decline with advancing age. However, given the conflicting findings regarding sex differences in postural control among older adults (Nakagawa et al., 2017; Šarabon et al., 2022), no directional hypothesis was formulated for sex-related effects or their interactions with age and sensory task difficulty. Examining these factors under systematically manipulated sensory conditions is therefore critical for disentangling age- and sex-specific strategies of multisensory balance control.

## 2 Materials and methods

### 2.1 Study sample

A total of 250 community-dwelling adults were recruited, comprising 50 younger adults (25–40 years; 25 males, 25 females) and 200 older adults (60–80 years; 100 males, 100 females). The older cohort was further stratified into four age groups at 5-year intervals (60–65, 66–70, 71–75, and 76–80 years), with 50 participants (25 males, 25 females) in each group. All participants were selected based on *a priori* inclusion and exclusion criteria (Table 1). The required sample size was estimated using G*Power software (version 3.1) for an F-test (within–between interactions). The calculation was based on an α error probability of 0.05, a statistical power (1–β) of 0.80, and an effect size of f = 0.25. This effect size was derived from the reported group differences in center of pressure (COP) velocity under sensory-challenged conditions in a previous study by Shams et al. (2020a), which assessed balance performance using a comparable experimental paradigm. The age range of 25–40 years was selected to represent young adulthood, a period of relatively stable physiological function and peak sensory-motor integration, serving as a robust baseline for age-related comparisons. Participants aged 40–60 years were intentionally excluded to create a clearer distinction between young and older cohorts and to accentuate balance differences attributable to advanced aging rather than gradual midlife transitions. This decision was informed by evidence indicating that postural control and multisensory integration remain relatively stable across much of midlife, with only gradual and heterogeneous changes observed during this period (Roman-Liu, 2018; Era et al., 2006; Hageman et al., 1995). In contrast, pronounced age-related declines in balance performance typically emerge after approximately 60 years of age, particularly under conditions that challenge sensory integration and require effective sensory reweighting (Jones and Noppeney, 2021; Sturnieks et al., 2008). Lifespan and cross-sectional analyses further demonstrate minimal deterioration in balance performance across early and middle adulthood, whereas substantial impairments become evident in later adulthood, especially when visual or somatosensory inputs are degraded (Era et al., 2006; Šarabon et al., 2022). Consequently, excluding the 40–60 age range minimized heterogeneity associated with midlife physiological transitions and enabled a more robust comparison between young adults and older cohorts exhibiting more established age-related deficits in multisensory balance control. Notably, the use of 60 years as a threshold to define older populations is well established in balance and aging research, and similar age stratification approaches have been widely adopted in both classical and contemporary studies of postural control in aging (Roman-Liu, 2018; Palazzo et al., 2021; Hageman et al., 1995; Sturnieks et al., 2008; Lehmann et al., 2025). This design allows for a more pronounced examination of the differential effects of advanced aging (60–80 years) on sensory-integrated balance control.

**TABLE 1:** Inclusion and exclusion criteria.

Category	Inclusion criteria	Exclusion criteria
Age	- Elderly: 60–80 years - Adults: 25–40 years	- Age outside the specified range (below 25 or above 80)
Height Body Mass, BMI	- Height, weight, body mass, and BMI in accordance with the iranian population for the relevant age/sex group - BMI calculated as body mass (kg)/height (m^2^) - No significant differences in height, weight, body mass, and BMI between participants in the same age/sex group	- Significant deviations in height, weight, body mass, or BMI from the normal curves for the respective age/sex group
Physical health	- General physical health	- History of fractures in the upper or lower limbs affecting balance - History of any physical damage affecting postural control
Mental health	- General mental health - Mini-mental state examination score of 24 or higher (for elderly participants)	- Neurological diseases
Physical activity & exercise history	- History of regular physical activity at moderate intensity or vigorous intensity, based on self-reported physical activity questionnaire	- Absence of regular physical activity and exercise
Sensory systems	- Natural or refined vision (with glasses or lenses)	- History of diseases affecting sensory systems
Medication	- Not using sleep medications	- Usage of sleep medications
Other	- No neurological diseases - No history of physical damage affecting postural control	​

BMI: body mass index.

### 2.2 Inclusion and exclusion criteria

Inclusion and exclusion criteria as shown in Table 1. The participant’s health status was evaluated with the general practitioner and geriatrician.

### 2.3 Materials

The CDP system is a quantitative and reliable method for assessing postural control and standing balance. The CDP system, particularly the SOT, has demonstrated good to excellent reliability and established validity for the assessment of postural control across diverse adult populations. Previous studies have reported test–retest intraclass correlation coefficients (ICCs) typically exceeding 0.75, with coefficients of variation generally falling within acceptable ranges (approximately 5%–15%), indicating a high level of measurement consistency (Ford-Smith et al., 1995; Cohen et al., 1993; Wrisley et al., 2007; Whitney et al., 2011). In clinical populations, including individuals with multiple sclerosis, SOT outcomes have shown ICC values ranging from 0.70 to 0.90 and have successfully differentiated participants according to disability severity, providing evidence of discriminant validity (Hebert and Manago, 2017). Furthermore, normative investigations in healthy adults have demonstrated moderate to good repeatability of SOT and related CDP measures (Trueblood et al., 2018). Collectively, these psychometric properties support the use of CDP as a robust and reliable tool for quantifying multisensory contributions to postural control in both healthy adults and clinical or aging populations.

This system is one of the most advanced for investigating center of pressure (COP) variables and man (Hebert and Manago, 2017) ipulating the sensory systems effective in postural control (Cumberworth et al., 2007; Ferber-Viart et al., 2007). The system’s SOT, which was used in this research, has demonstrated good-to-excellent test-retest reliability in previous studies, with ICC values often exceeding 0.75 (Ford-Smith et al., 1995; Cohen et al., 1993; Wrisley et al., 2007; Whitney et al., 2011). The SOT evaluates the performance of the sensory systems in postural control through six conditions. In the first three conditions, the force plates are fixed, and in the other three, they move in the anterior–posterior direction. In Condition 1 (baseline), the participant is placed on the system with all sensory information available. In Condition 2, the participant is tested with a blindfold (eliminating the visual system). In Condition 3, the eyes are open but the visual environment is sway-referenced, leading to incorrect visual arrays (Shams et al., 2020a; Cumberworth et al., 2007). In the 4 to 6 conditions, the force plates are also sway-referenced, making proprioceptive information inaccurate. This requires participants to rely on visual and/or vestibular information to maintain their balance, overcoming the inaccurate proprioceptive signals through compensatory mechanisms. Shams et al. (2020a) stated that the COP variables in stance conditions on the force plate with foam were significantly correlated with SOT conditions. In condition 4, the information from the visual and vestibular systems is evaluated. In condition 5, the participant eyes are blindfolded and information from the vestibular system was tested. In condition 6, the vestibular system information is evaluated and incorrect visual arrays are presented. The duration of each test condition is 20 s and each condition will be repeated thrice (Shams et al., 2020a; Paniccia et al., 2018). Each person completed testing as shown in Table 2.

**TABLE 2:** Sensory organization test conditions.

Test condition	Eyes	Surroundings	Platform	Sensory system used	Disadvantaged sensory system
1	Open	Fixed	Fixed	Somatosensory	—
2	Closed	—	Fixed	Somatosensory	Visual
3	Open	Sway referenced	Fixed	Somatosensory	Visual
4	Open	Fixed	Sway referenced	Visual	Somatosensory
5	Closed	—	Sway referenced	Vestibular	Somatosensory/Visual
6	Open	Sway referenced	Sway referenced	Vestibular	Somatosensory/Visual

Adapted from the Balance Manager Clinical Operation Guide, NeuroCom.

### 2.4 Procedure

In the first step, the distance between the two legs for placement was standardized based on the width of the pelvis, which was equal to 50% of hip to hip distance (Anterior-Superior Iliac Spine). The distance obtained for each person was also used at all the test stages as a placement criterion (Shams et al., 2020a; Peterson et al., 2006). The test environment had adequate lighting and ventilation, and a suitable temperature for performing the tests. Moreover, during the course of the research, there was complete silence, and the same conditions were observed during the tests for all the participants. Each participant was placed on the force plates of the CDP system with bare feet after receiving the necessary notes on how the test was performed (Shams et al., 2020a; Peterson et al., 2006). The variables used in this study included the Overall COP velocity and displacements. In all of the test conditions, the COP variables were measured, stored, and analyzed in the next steps. All the participants completed three tests in each of the situations and the mean of the numbers obtained was analyzed (Peterson et al., 2006; Choy et al., 2003). After completing the test conditions, the COP variables were analyzed by the Neurocom System Data Analyzer software and stored in a notepad file. For analyzing the COP mean amplitude, first the standard deviation of the amplitude was calculated in the anterior–posterior direction and medial-lateral direction. Then, the mean values obtained were considered as the mean oscillation amplitude of the COP. The formulas were also used to analyze the overall velocity of COP (Shams et al., 2020a; Prieto et al., 1996; Winter et al., 1996; Moghadam et al., 2011).

### 2.5 Statistics

For data analysis, descriptive statistics (means, standard deviations) were calculated after confirming data normality with the Shapiro-Wilk test. An exploratory multivariate analysis of variance (MANOVA) was first conducted to examine overall multivariate patterns and potential associations among age group, sex, and sensory conditions across the set of dependent balance variables. This multivariate approach was used to identify whether combined postural control outcomes differed across groups before proceeding to univariate analyses. Subsequently, a 5 (Age Group: 25–40, 60–65, 66–70, 71–75, 76–80 years) × 2 (sex) × 6 (sensory conditions) repeated-measures analysis of variance (ANOVA) was conducted to investigate the effects of sensory conditions (within-subject factor) on balance control, with age and sex as between-subject factors. The assumptions of normality and sphericity were verified. Post-hoc analyses were performed using Tukey’s HSD test. Effect sizes were initially obtained as partial eta-squared (*η*_p_^2^) directly from the ANOVA and MANOVA outputs to quantify the magnitude of significant main and interaction effects. For ease of interpretation and comparison across effects, partial eta-squared values were subsequently transformed into Cohen’s *f* using the formula *f* = √(*η*_p_^2^/(1 − *η*_p_^2^)), and then converted to Cohen’s *d* (*d* = 2*f*) (Kim, 2016; Cohen, 1988). Cohen’s *d* values were interpreted according to conventional thresholds, with values of 0.2 indicating a small effect, 0.5 a medium effect, and 0.8 or greater a large effect (Sullivan and Feinn, 2012). The alpha level was set at p < 0.05. All analyses were performed using SPSS (Version 26).

## 3 Results

**FIGURE 1:**
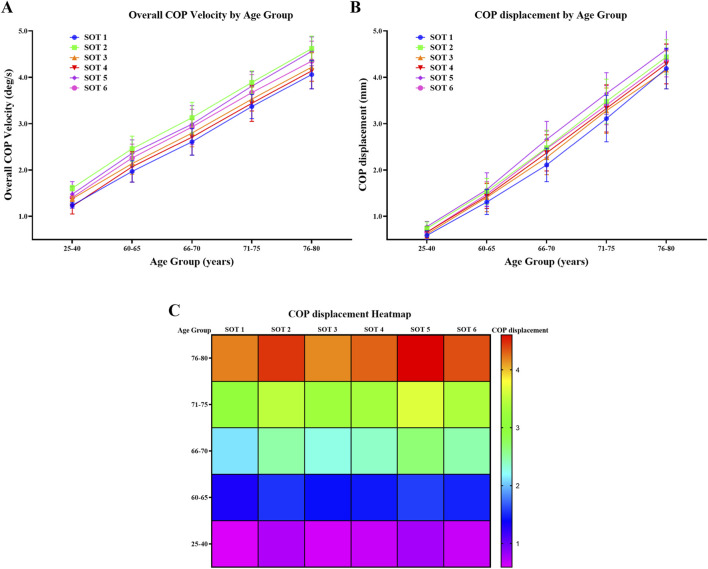
Age-related changes in postural control under different sensory conditions **(A)** Changes in overall center of pressure (COP) velocity (deg/s) across age groups under six Sensory Organization Test (SOT) conditions. **(B)** Age-related changes in COP displacement (mm) across the same age groups and SOT conditions. **(C)** Heatmap illustrating mean COP displacement (mm) across age groups and SOT conditions. Values are expressed as mean ± SD. SOT: Sensory Organization Test; COP: center of pressure.

The Shapiro-Wilk test indicated that the data for all outcome measures were normally distributed (p > 0.05). Mean and standard deviations for age, height, mass, and BMI of the individuals are presented in Table 3. No statistically significant between-group differences were found for anthropometrics (height, mass, and BMI). Tables 4, 5, and Figure 1 present the mean and standard deviation of the overall COP velocity and displacement variables.

**TABLE 3:** Descriptive results of age, height, body mass, and BMI.

Age group	Sex	n	Age (year)	Height (m)	Body mass (kg)	BMI (kg/m^2^)
25–40	Males	25	29.20 ± 8.30	1.70 ± 0.18	73.55 ± 3.50	26.65 ± 3.31
	Females	25	33.80 ± 6.50	1.68 ± 0.06	71.85 ± 5.50	24.25 ± 1.11
	Total	50	31.50 ± 7.40	1.69 ± 0.12	72.70 ± 4.50	25.45 ± 2.21
60–65	Males	25	64.05 ± 2.00	1.67 ± 0.13	73.50 ± 5.10	27.34 ± 1.64
	Females	25	62.15 ± 1.00	1.63 ± 0.07	69.50 ± 3.10	25.20 ± 1.40
	Total	50	63.10 ± 1.50	1.65 ± 0.10	71.50 ± 4.10	26.27 ± 1.52
66–70	Males	25	68.30 ± 1.10	1.65 ± 0.17	69.30 ± 6.60	26.66 ± 4.15
	Females	25	68.10 ± 1.30	1.61 ± 0.09	67.10 ± 4.80	24.68 ± 2.35
	Total	50	68.20 ± 1.20	1.63 ± 0.13	68.20 ± 5.70	25.67 ± 3.25
71–75	Males	25	72.40 ± 1.00	1.68 ± 0.11	76.70 ± 6.30	27.61 ± 3.04
	Females	25	74.20 ± 2.00	1.66 ± 0.07	72.30 ± 4.10	25.81 ± 1.64
	Total	50	73.30 ± 1.50	1.67 ± 0.09	74.50 ± 5.20	26.71 ± 2.34
76–80	Males	25	77.70 ± 1.80	1.62 ± 0.20	68.20 ± 5.20	27.60 ± 2.47
	Females	25	79.10 ± 0.60	1.56 ± 0.08	66.40 ± 3.80	25.66 ± 2.69
	Total	50	78.40 ± 1.20	1.59 ± 0.14	67.30 ± 4.50	26.63 ± 2.58

BMI: body mass index.

**TABLE 4:** Means and standard deviations of overall COP velocity (deg/s) for the sensory organization test, by age group and sex.

Age group	Sex	n	SOT 1	SOT 2	SOT 3	SOT 4	SOT 5	SOT 6
25–40	Males	25	1.23 ± 0.05	1.61 ± 0.06	1.37 ± 0.06	1.24 ± 0.17	1.44 ± 0.29	1.41 ± 0.24
	Females	25	1.25 ± 0.06	1.61 ± 0.05	1.38 ± 0.06	1.20 ± 0.16	1.51 ± 0.26	1.42 ± 0.22
	Total	50	1.24 ± 0.06	1.61 ± 0.06	1.38 ± 0.06	1.22 ± 0.17	1.48 ± 0.27	1.41 ± 0.23
60–65	Males	25	1.98 ± 0.21	2.47 ± 0.23	2.15 ± 0.22	2.10 ± 0.33	2.40 ± 0.22	2.27 ± 0.30
	Females	25	1.96 ± 0.25	2.45 ± 0.31	2.14 ± 0.25	2.03 ± 0.34	2.32 ± 0.35	2.25 ± 0.30
	Total	50	1.97 ± 0.23	2.46 ± 0.27	2.14 ± 0.23	2.07 ± 0.33	2.36 ± 0.29	2.26 ± 0.30
66–70	Males	25	2.60 ± 0.29	3.15 ± 0.34	2.78 ± 0.27	2.70 ± 0.40	3.01 ± 0.41	2.87 ± 0.31
	Females	25	2.61 ± 0.29	3.11 ± 0.32	2.83 ± 0.33	2.72 ± 0.39	2.98 ± 0.40	2.99 ± 0.44
	Total	50	2.61 ± 0.29	3.13 ± 0.33	2.80 ± 0.30	2.71 ± 0.39	2.99 ± 0.40	2.93 ± 0.38
71–75	Males	25	3.41 ± 0.25	3.90 ± 0.26	3.57 ± 0.24	3.52 ± 0.39	3.84 ± 0.33	3.62 ± 0.39
	Females	25	3.33 ± 0.27	3.87 ± 0.25	3.48 ± 0.28	3.37 ± 0.38	3.79 ± 0.30	3.72 ± 0.38
	Total	50	3.37 ± 0.26	3.89 ± 0.25	3.53 ± 0.26	3.44 ± 0.39	3.81 ± 0.31	3.67 ± 0.39
76–80	Males	25	3.89 ± 0.30	4.56 ± 0.30	4.04 ± 0.30	3.92 ± 0.35	4.47 ± 0.35	4.15 ± 0.43
	Females	25	4.24 ± 0.19	4.68 ± 0.23	4.40 ± 0.18	4.35 ± 0.29	4.64 ± 0.23	4.56 ± 0.32
	Total	50	4.06 ± 0.31	4.62 ± 0.27	4.22 ± 0.31	4.14 ± 0.39	4.56 ± 0.31	4.35 ± 0.43

Values are given as mean ± SD. n: number of participants in each group, SOT: condition.

**TABLE 5:** Means and standard deviations of COP displacement (mm) for the sensory organization test, by age group and sex.

Age group	Sex	n	SOT 1	SOT 2	SOT 3	SOT 4	SOT 5	SOT 6
25–40	Males	25	0.59 ± 0.11	0.76 ± 0.13	0.60 ± 0.10	0.65 ± 0.12	0.78 ± 0.10	0.66 ± 0.11
	Females	25	0.58 ± 0.10	0.72 ± 0.15	0.64 ± 0.09	0.68 ± 0.12	0.78 ± 0.11	0.67 ± 0.13
	Total	50	0.59 ± 0.10	0.74 ± 0.14	0.62 ± 0.10	0.66 ± 0.12	0.78 ± 0.11	0.66 ± 0.12
60–65	Males	25	1.33 ± 0.19	1.51 ± 0.26	1.37 ± 0.27	1.44 ± 0.27	1.55 ± 0.32	1.46 ± 0.23
	Females	25	1.28 ± 0.33	1.58 ± 0.29	1.46 ± 0.36	1.43 ± 0.28	1.61 ± 0.41	1.49 ± 0.31
	Total	50	1.31 ± 0.27	1.54 ± 0.28	1.41 ± 0.31	1.44 ± 0.27	1.58 ± 0.36	1.48 ± 0.27
66–70	Males	25	2.10 ± 0.36	2.55 ± 0.39	2.27 ± 0.38	2.33 ± 0.38	2.71 ± 0.40	2.47 ± 0.37
	Females	25	2.12 ± 0.38	2.41 ± 0.35	2.28 ± 0.37	2.41 ± 0.40	2.62 ± 0.38	2.45 ± 0.39
	Total	50	2.11 ± 0.36	2.48 ± 0.38	2.27 ± 0.37	2.37 ± 0.39	2.66 ± 0.39	2.46 ± 0.38
71–75	Males	25	3.17 ± 0.53	3.51 ± 0.49	3.36 ± 0.52	3.34 ± 0.56	3.64 ± 0.45	3.41 ± 0.41
	Females	25	3.05 ± 0.47	3.45 ± 0.47	3.21 ± 0.46	3.32 ± 0.47	3.65 ± 0.45	3.39 ± 0.43
	Total	50	3.11 ± 0.50	3.48 ± 0.48	3.28 ± 0.49	3.33 ± 0.51	3.65 ± 0.45	3.40 ± 0.42
76–80	Males	25	4.09 ± 0.51	4.34 ± 0.39	4.14 ± 0.49	4.24 ± 0.54	4.47 ± 0.47	4.30 ± 0.41
	Females	25	4.29 ± 0.34	4.54 ± 0.33	4.19 ± 0.32	4.33 ± 0.30	4.74 ± 0.47	4.42 ± 0.29
	Total	50	4.19 ± 0.44	4.44 ± 0.37	4.16 ± 0.41	4.29 ± 0.43	4.60 ± 0.42	4.36 ± 0.35

Values are given as mean ± SD. n: number of v in each group, SOT: condition.

### 3.1 Overall COP velocity

A 5 (age) × 2 (sex) × 6 (sensory conditions) repeated-measures ANOVA was conducted to evaluate the effects of age, sex, and sensory condition on balance performance, as measured by total COP velocity. Greenhouse-Geisser corrections were applied where sphericity was violated (Mauchly’s W = 0.129, p < 0.001). The analysis revealed a significant main age effect (F_(4,240)_ = 1281, p < 0.001, *d* = 9.21, large effect), indicating that balance performance declined significantly with increasing age. Post-hoc pairwise comparisons with Bonferroni correction confirmed that all five age groups (25–40, 60–65, 66–70, 71–75, and 76–80 years) differed significantly from each other (p < 0.001 for all comparisons), with the oldest group exhibiting the poorest balance (highest velocity) and the youngest group the best (lowest velocity). A significant main sensory condition effect was found (F_(5,1200)_ = 337.26, p < 0.001, *d* = 2.37, large effect). Post-hoc tests indicated that nearly all pairwise comparisons between the six sensory conditions were statistically significant (p < 0.05), demonstrating that altering sensory input substantially impacts postural stability. No statistically significant main effect of sex was found (F_(1,240)_ = 2.69, p = 0.102, *d* = 0.21, small effect), indicating that, on average across all age groups and sensory conditions, males and females did not differ significantly in overall balance performance.

Significant age × group interactions were found (F_(20,1200)_ = 3.68, p < 0.001, *d* = 0.5, medium effect), indicating that the effect of sensory condition on balance depended on age, with older adults showing more adverse effects caused by challenging sensory conditions than younger adults.

Conditions × sex interactions were significant (F_(5,1200)_ = 3.69, p = 0.003, *d* = 0.25, small effect), suggesting that males and females responded differently to specific sensory manipulations. The age × sex interaction was significant (F_(4,240)_ = 5.15, p = 0.001, *d* = 0.59, medium effect). Simple effects analysis revealed that sex differences were most pronounced in the oldest age group (76–80 years), where females demonstrated poorer balance (higher velocity) than males, a difference that was negligible in younger groups. Finally, the three-way age × sex × conditions interaction was also significant (F_(20,1200)_ = 2.19, p = 0.002, *d* = 0.38, small to approaching medium effect), confirming that the complex interplay between age, sex, and sensory conditions collectively influences balance performance.

Box’s M test indicated a violation of the assumption of homogeneity of covariance matrices (p < 0.001), so Pillai’s Trace was used as the robust test statistic. The MANOVA revealed a significant multivariate main effect for age group (Pillai’s Trace = 1.080, *F*_(24,952)_ = 14.68, p < 0.001, *d* = 1.22, large effect), indicating that age had a large and significant effect on the combined balance measures. There was also a significant multivariate main effect for sex (Pillai’s Trace = 0.055, *F*_(6,235)_ = 2.276, p = 0.037, *d* = 0.48, Small-to-medium (borderline) effect), though the effect size was small. This suggests that while the primary ANOVA showed no overall sex effect, there are subtle, condition-specific differences when considering all sensory conditions simultaneously. The age group × Sex interaction was also significant (Pillai’s Trace = 0.223, *F*_(24,952)_ = 2.338, p < 0.001, *d* = 0.49, Small-to-medium (borderline) effect). Follow-up univariate ANOVAs were conducted for each dependent variable, with Bonferroni-adjusted pairwise comparisons for age group. Age group had a significant main effect on all six balance measures (all p < 0.001), with very large effect sizes (Cohen’s d ranging from 6.6 to 8.91, all large effects). Post-hoc tests confirmed that all five age groups differed significantly from each other on every condition (p < 0.001), with balance performance declining (velocity increasing) progressively with age. Sex had a significant univariate effect on three of the six balance measures: total Velocity in condition 3: *F*_(1,240)_ = 4.461, p = 0.036, *d* = 0.27, small effect, total Velocity in condition 6: *F*_(1,240)_ = 9.051, p = 0.003, *d* = 0.39, small to approaching effect, total Velocity in condition 1 showed a non-significant trend: *F*_(1, 240)_ = 3.809, p = 0.052, *d* = 0.26, small effect, No significant effects were found for Velocities in condition 2, 4, or 5 (all p > 0.05). Consistent with the significant interaction in the primary ANOVA, *post hoc* inspection revealed that females tended to have higher velocities (worse balance) than males in some conditions, particularly in the oldest age groups.

The age group × sex interaction was significant for: total velocity in condition 1: *F*_(4,240)_ = 6.590, p < 0.001, *d* = 0.66, medium to large effect, total velocity in condition 3: *F*_(4,240)_ = 6.955, p < 0.001, *d* = 0.68, medium to large effect, total velocity in condition 4: *F*_(4,240)_ = 6.373, p < 0.001, *d* = 0.65, medium to large effect, total velocity in condition 6: *F*_(4,240)_ = 3.747, p = 0.006, *d* = 0.49, small-to-medium (borderline) effect, The interaction was not significant for Velocities in condition 2 or 5. A series of one-way analyses of variance (ANOVAs) was conducted to evaluate the effect of age group (5 levels: 25–40, 60–65, 66–70, 71–75, 76–80 years) on balance performance, measured by center-of-pressure velocity under six different sensory conditions. Post-hoc comparisons were performed using the Bonferroni correction. There was a statistically significant effect of age group on balance performance for all six sensory conditions: condition 1: *F*_(4,245)_ = 1069, p < 0.001, *d* = 8.37, large effect, condition 2: *F*_(4, 245)_ = 1131, p < 0.001, *d* = 8.63, large effect, condition 3: *F*_(4,245)_ = 1035, p < 0.001, *d* = 8.21, large effect, condition 4: *F*_(4,245)_ = 600.61, p < 0.001, *d* = 6.25, large effect, condition 5: *F*_(4,245)_ = 868.70, p < 0.001, *d* = 7.52, large effect, condition 6: *F*_(4,245)_ = 675.87, p < 0.001, *d* = 6.65, large effect. Bonferroni *post hoc* tests revealed that all pairwise comparisons between age groups were statistically significant (p < 0.001) for every sensory condition. Balance performance systematically worsened (velocity increased) with each successive age group. A series of independent samples t-tests was conducted to compare balance performance, as measured by center-of-pressure velocity under six sensory conditions, between males and females. Descriptive statistics indicated that females had slightly higher mean velocity scores (indicating worse balance) than males across all six conditions, though the differences were small (see Table 4). Levene’s test indicated that the assumption of homogeneity of variances was met for all conditions (p > 0.05). The t-tests revealed that there were no statistically significant differences between males and females on any of the six conditions.

### 3.2 COP displacement

A 5 (age Group) × 2 (sex) × 6 (sensory condition) repeated-measures ANOVA was conducted to evaluate the effects of age, sex, and sensory manipulation on balance performance, as measured by the standard deviation of sway displacement in medial-lateral and anterior–posterior directions. Mauchly’s test indicated a violation of the sphericity assumption for the within-subjects factor (W = 0.867, p = 0.002); therefore, the Greenhouse-Geisser corrected values (ε = 0.944) were used for interpretation. However, for clarity, the original degrees of freedom and significance levels are reported below, as the correction did not alter the interpretation of any effects. A significant main effect of age was found, *F*_(4,240)_ = 1465.00, p < 0.001, *d* = 9.93, large effect). Post-hoc pairwise comparisons with Bonferroni adjustment revealed that all five age groups were significantly different from each other (p < 0.001), with postural sway decreasing significantly with each successive younger age group (76–80 > 71–75 > 66–70 > 60–65 > 25–40 years). A significant main effect of sensory condition was also found (*F*_(5,1200)_ = 85.96, p < 0.001, *d* = 1.2, large effect). Post-hoc tests indicated that performance varied significantly across most of the six sensory conditions. Condition 5 was associated with the greatest sway (poorest performance), which was significantly higher than all other conditions (p < 0.001). Condition 1 was associated with the least sway, which was significantly lower than all other conditions (p < 0.05 for all comparisons). The main effect of sex was not statistically significant (*F*_(1, 240)_ = 0.531, p = 0.467, *d* = 0.09, very small effect), indicating that males and females did not differ significantly in their overall postural sway. A significant two-way interaction was observed between age and sensory condition (*F*_(20,1200)_ = 3.18, p < 0.001, *d* = 0.46, Small-to-medium (borderline) effect). This indicates that the effect of sensory manipulation on postural sway was not uniform across age groups. Older age groups exhibited a more pronounced increase in sway in response to the more challenging sensory conditions compared to younger groups. The age × sex interaction was not significant (*F*_(4,240)_ = 1.133, p = 0.341, *d* = 0.28, small effect). Similarly, the sex × sensory condition interaction was not significant (*F*_(5,1200)_ = 0.324, p = 0.899, *d* = 0.06, very small effect). Finally, the three-way age × sex × sensory condition interaction was also not significant (*F*_(20,1200)_ = 1.140, p = 0.301, *d* = 0.28, small effect).

A 5 (age group) × 2 (sex) MANOVA was conducted to assess the effects of age and sex on postural sway across six different sensory conditions. The dependent variables were the standard deviation of sway displacement in medial-lateral and anterior–posterior directions under each condition. Box’s M test was significant (p < 0.001), indicating a violation of the homogeneity of covariance matrices assumption. Therefore, the more robust Pillai’s Trace statistic was used for interpreting multivariate effects. A significant multivariate main effect was found for Age Group, Pillai’s Trace = 1.094, *F*_(24, 952)_ = 14.94, p < 0.001, *d* = 1.23, large effect. This indicates that age groups differed significantly on a linear combination of the six sway measures. The multivariate main effect for Sex was not significant, Pillai’s Trace = 0.010, *F*_(6,235)_ = 0.385, p = 0.888, *d* = 0.2, small effect, indicating no overall difference between males and females across the sway conditions. The Age Group × Sex interaction was also not significant, Pillai’s Trace =0 .094, *F*_(24, 952)_ = 0.953, p = 0.527, *d* = 0.31, small to approaching medium effect. Significant univariate main effects for Age Group were found for all six dependent variables (all p < 0.001), with very large effect sizes (Cohen’s *d* ranging from 7.40 to 8.37, all large effects). Post-hoc pairwise comparisons with Bonferroni correction revealed that all five age groups were significantly different from each other on every sway measure (all p < 0.001). Sway amplitude was highest in the oldest group (76–80 years) and decreased progressively with each younger age group, with the youngest group (25–40 years) exhibiting the smallest sway.

No significant univariate main effects for sex were found for any of the six sway measures (all p > 0.05), confirming the multivariate result that balance performance did not differ between males and females. The age group × sex interaction was not significant for any of the dependent variables (all p > 0.05). A series of one-way analyses of variance (ANOVAs) was conducted to evaluate the effect of age group (5 levels: 25–40, 60–65, 66–70, 71–75, 76–80 years) on postural sway variability, measured by the standard deviation of sway displacement in anterior–posterior and medial-lateral directions under six sensory conditions. Post-hoc comparisons were performed using the Bonferroni correction. There was a statistically significant effect of Age Group on postural sway variability for all six sensory conditions: Condition 1: *F*_(4,245)_ = 814.92, p < 0.001, *d* = 7.18, large effect, Condition 2: *F*_(4,245)_ = 927.34, p < 0.001, *d* = 7.78, large effect, Condition 3: *F*_(4,245)_ = 827.03, p < 0.001, *d* = 7.35, large effect, Condition 4: *F*_(4,245)_ = 888.46, p < 0.001, *d* = 7.59, large effect, Condition 5: *F*_(4,245)_ = 877.65, p < 0.001, *d* = 7.59, large effect, Condition 6: *F*_(4,245)_ = 1057, p < 0.001, *d* = 8.29, large effect. The effect sizes were all above 0.92, indicating that age group accounts for over 92% of the variance in postural sway variability across all conditions. Bonferroni *post hoc* tests revealed that all pairwise comparisons between age groups were statistically significant (p < 0.001) for every sensory condition. Postural sway variability systematically increased with each successive age group. The oldest group (76–80 years) exhibited the highest sway variability, while the youngest group (25–40 years) showed the lowest. A series of independent samples t-tests was conducted to compare balance performance, as measured by sway displacement in medial-lateral and anterior–posterior directions under six sensory conditions, between males and females. Descriptive statistics indicated that females had slightly higher mean velocity scores (indicating worse balance) than males across all six conditions, though the differences were small (see Table 5). Levene’s test indicated that the assumption of homogeneity of variances was met for all conditions (p > 0.05). The t-tests revealed that there were no statistically significant differences between males and females on any of the six conditions.

## 4 Discussion

This study found that sensory conditions significantly affected balance control across all measures, with greater challenges leading to poorer stability. Age was a key determinant of balance performance, with the oldest group (76–80 years) consistently showing greater instability across all conditions and balance measures. These findings are consistent with previous research showing age-related decline in postural control due to decreased sensory integration and motor responses in older adults. Interestingly, while no main effect of sex was found, significant interactions revealed that sex had context-dependent effects on balance, with age-related sex differences observed in specific measures. These results suggest that while aging has a profound impact on balance, the influence of sex is more nuanced and becomes apparent primarily in advanced age. Our results demonstrate that elderly individuals from different age groups had different COP variables in terms of the availability of all sensory information, showing that the postural control decreased with age. This reduction and variable patterns created among the elderly can be attributed to a decrease in the sensitivity and function of the sensory systems involved in the postural control (Woollacott and Shumway-Cook, 2002; Ruhe et al., 2010; Freitas et al., 2005). The particularly large decline in performance in SOT conditions 5 and 6, which challenge proprioception and vision, strongly points to a significant degradation of vestibular function and sensory reweighting capacity with age (Birnbaum et al., 2017; Faraldo-García et al., 2016).

While the SOT is a standardized laboratory assessment (De Pasquale et al., 2025), its conditions are directly analogous to common, challenging scenarios in daily life that are strongly associated with falls. For example, SOT conditions with eyes closed (Conditions 2 & 5) simulate navigating in low-light conditions, at night, or when visual attention is diverted (Müjdeci et al., 2012). Conditions with sway-referenced support (Conditions 4–6) mimic the experience of standing on a moving bus, walking on soft or uneven terrain like grass or gravel, or maintaining balance on a slippery surface (Sung and Rowland, 2024). The most challenging condition (SOT 5), which removes both accurate vision and proprioception, parallels situations like standing up in a dark bathroom or finding footing on an unstable surface without a reliable visual reference (Müjdeci et al., 2012). The pronounced and progressive decline in performance across these conditions, especially in the oldest age groups, provides a quantifiable link between impaired sensory integration and increased biomechanical instability—the direct precursor to a fall (Sung and Rowland, 2024). Therefore, the significant age × sensory condition interaction observed is not merely a laboratory phenomenon; it reflects a critical degradation of the balance system’s functional reserve needed to safely manage the complex and often unpredictable sensory environments of daily living. Furthermore, the identification of the oldest females as particularly vulnerable under these challenging conditions highlights a key demographic for whom targeted fall risk assessment—using tools like the SOT—and preventative intervention are most crucial. Generally, as age increases from childhood to adulthood, a major organization process occurs at all levels of brain structure and functions. The results of the studies indicate that the brain’s sensory–motor cortex map shows a lot of changes. This organization of the cerebral cortex is parallel to the increase in the function of the sensory systems and ultimately leads to an increased performance of the postural control system (Chen et al., 2007). In old age, the integration and reliability of sensory information progressively decline, leading to impaired balance and postural control and, consequently, an increased risk of falls.

The emergence of sex differences specifically in the oldest cohort (76–80 years), where females exhibited greater COP velocity, aligns with literature on sex-specific aging trajectories. Several interacting physiological and neurological mechanisms may explain this finding. First, a more rapid decline in muscle mass and strength (sarcopenia) and power in women compared to men, particularly in the lower limbs, can directly impair the biomechanical capacity to execute rapid and forceful postural corrections. Second, sex hormones, particularly the sharp decline in estrogen during menopause, have been linked to changes in neuromuscular function, proprioceptive acuity, and potentially vestibular processing, which may compromise sensory integration under demanding balance conditions. Third, structural and functional differences in the aging brain, such as differential rates of cortical thinning or declines in white matter integrity in areas related to sensorimotor integration, could affect the central processing and reweighting of sensory information. Finally, subtle, subclinical differences in the prevalence of peripheral neuropathies affecting somatosensory feedback from the feet, or age-related vestibular hypofunction, may disproportionately affect older women. The convergence of these factors in the eighth decade of life likely underlies the observed vulnerability, where the sensory-challenging SOT conditions unmask a latent postural control deficit in the oldest females that is not evident in younger groups or under simple stance conditions. Vestibular information is one of the most important sets of information in the postural control of individuals; therefore, sending this information to higher brain centers can lead to optimal postural control. This system has an anatomic structure and a very complex physiological function, which plays an important role in many human functions. Although the vestibular system is anatomically mature at the time of birth, the maturity process of its physiological and functional performance continues until the age of 14–15. Then, it declines significantly among the elderly (Chen et al., 2007; Shumway-Cook and Woollacott, 2012). By eliminating visual information, the COP variables of the elderly changed and postural control decreased. In general, visual inputs are important information sources for postural control and they directly affect that of the individuals. Visual inputs provide information related to the position and movement of the head, considering environmental objects, and are used as a very important reference as the visual system transmits information on peripheral and central vision to the cortex and then to the vision areas (Shumway-Cook and Woollacott, 2012). Therefore, although the visual system is not the only source of sensory information, it is important for postural control (Shumway-Cook et al., 1997).

In the position of presenting inappropriate visual arrays, older people show more fluctuations in this condition than adults. This is also due to their inability to process information from the plantar and ankles, which results in excessive reliance on visual inputs in postural control (Chen et al., 2007). According to studies, after the damage of the vestibular system or the reduction of proprioception information, the older adults can still use the mechanisms of the visual system with an acceptable performance to maintain postural control. Therefore, when individuals perceive inaccurate information, the amount of body fluctuation will be much higher. So, when people close their eyes, the rate of postural fluctuations rises from 22%–56% (Shumway-Cook and Woollacott, 2012; Shumway-Cook et al., 1997). During old age, the integration of the information obtained by the sensory systems, especially through proprioception, is reduced so that people will not be able to have postural control similar to that of adults without the use or availability of visual information. Reduced postural control of the elderly compared to adults in terms of the deletion of proprioception information happens because, after the information and inputs of the visual system, the proprioception sensory inputs of the legs are very important for controlling the posture. According to the studies, the reduction of sensory inputs from the lower body, including the localized ischemic conditions, leads to an increase in the COP variables in the standing position (Macpherson et al., 1997; Jeka et al., 2000).

The elderly individuals experience a high drop in the control of their posture when the visual and the vestibular system information are being removed simultaneously (Cohen et al., 1996). Children and older adults also show greater fluctuations in this situation than adults. This also happens due to their inability to process information from the plantar and ankles, which results in excessive reliance on the visual inputs in postural control (Kim et al., 2008). The proprioception inputs provide information on the orientation of different body parts relative to one another, as well as to the reliance level of the body. The sense of sight perceives the orientation of the eyes and head toward the objects around. Also, the vestibular system provides information on gravity, and on linear and angular accelerations in relation to space, but this system does not relate to the orientation of the surrounding objects. As a result, when visual and proprioception systems provide accurate information, the vestibular system plays a minor role in maintaining the postural control and balance among individuals (Prado et al., 2011).

Regarding the presentation of inappropriate visual arrays, the sensory weighting hypothesis suggests that the central nervous system changes the weight (or importance) of the input of sensory systems based on their accuracy ratio in the postural control navigation component (Roll et al., 1988). In this hypothesis, the central nervous system retrieves sensory conflicts (conditions, in which there is no agreement between sensory inputs) to change the weight associated with a sensory input in the postural control (Shumway-Cook et al., 1997). For example, when the visual information is not accurate, the proprioception sensory system information will be weighed and used (Prado et al., 2011; Roll et al., 1988). Another explanation for these results may be that older adults cannot effectively use the feed-forward control for their postural control and that they may use the feedback control. As researchers stated that, when precise control of the center of pressure is required, the person cannot rely on inactive control, in which the changes in the position cause the regulator response. Therefore, when the feedback is removed, people are forced to use the feed-forward control, which is able to adjust the pressure center adequately (Kim et al., 2008; Prado et al., 2011; Roll et al., 1988; Shams et al., 2020b). Also, when the postural control is considered as two parallel processes—one continuous and the other related to a response stimulus—we can argue that older adults depended more on the response stimulus process. The inability of the elderly to use the continuous process can once again relate to sensory and motor changes inflicted by increasing age.

Several limitations should be considered when interpreting the findings of this study. First, the study relied on a relatively simple classification of sex (male and female), which may not fully capture the spectrum of sex diversity and its potential impact on balance control. Future research could benefit from a more inclusive approach to sex categorization. Second, although the sensory conditions used in the study were designed to challenge postural control, they may not fully replicate real-world scenarios where balance is affected by more complex and dynamic environments. For instance, the static stance of the SOT does not incorporate the additional cognitive load of dual-tasking (e.g., talking while walking) or the dynamic whole-body coordination required for gait or obstacle avoidance. The findings might therefore be more reflective of controlled laboratory settings rather than practical, everyday situations.

Finally, this study focused on short-term balance measures under controlled conditions. Future studies could incorporate longitudinal designs to assess how balance control changes over time and in response to interventions, providing a more comprehensive understanding of age-related balance decline. Although normative aging is often associated with changes in stature and body composition, the lack of statistically significant differences in height, mass, and BMI across the five age groups in this sample likely reflects careful stratification and recruitment aimed at minimizing confounding anthropometric variability. Participants were screened to align with population norms for their respective age and sex groups in Iran, which may have reduced inter-group variance. Consequently, the observed balance differences are more directly attributable to age-related physiological and sensory changes rather than anthropometric factors.

## 5 Conclusion

This study provides clear evidence that balance control is significantly influenced by sensory conditions, age, and, to a lesser extent, sex. The findings reveal that older adults, particularly those aged 76–80 years, exhibit greater instability in all balance compared to younger individuals. These age-related differences in postural control were more pronounced under challenging sensory conditions, highlighting the deteriorating ability of older adults to integrate sensory inputs and maintain balance.

Highlighting the deteriorating ability of older adults to integrate sensory inputs and maintain balance. It is important to note that no generalized sex difference in balance control was observed across the entire sample. The significant age × sex interaction indicates that sex-related differences in postural stability are not universal but are specific to advanced age, emerging as a meaningful factor only in the oldest cohort (76–80 years). These findings indicate that the effect of sex is marginal compared to the dominant effect of age but identifies the oldest females as a high-risk subgroup. The results have important implications for fall prevention strategies and rehabilitation programs aimed at improving balance control in older adults. Given the significant decline in balance with age, particularly under sensory-challenging conditions, interventions should focus on enhancing sensory integration and compensatory mechanisms to mitigate age-related balance impairments. Future research should further investigate the underlying mechanisms driving these age- and sex-related differences in balance control and explore targeted interventions that could improve postural stability in older populations.

## Data Availability

The original contributions presented in the study are included in the article/supplementary material, further inquiries can be directed to the corresponding authors.
